# Generalisability and Cost-Impact of Antibiotic-Impregnated Central Venous Catheters for Reducing Risk of Bloodstream Infection in Paediatric Intensive Care Units in England

**DOI:** 10.1371/journal.pone.0151348

**Published:** 2016-03-21

**Authors:** Katie Harron, Quen Mok, Dyfrig Hughes, Berit Muller-Pebody, Roger Parslow, Padmanabhan Ramnarayan, Ruth Gilbert

**Affiliations:** 1 Institute of Child Health, University College London, 30 Guilford Street, London WC1 N 1EH, United Kingdom; 2 Paediatric Intensive Care Unit, Great Ormond Street Hospital, London, WC1N 3JH, United Kingdom; 3 Centre for Health Economics and Medicines Evaluation, Bangor University, Bangor, LL57 2PZ, United Kingdom; 4 Public Health England, 61 Colindale Avenue, London, NW9 5EQ, United Kingdom; 5 University of Leeds, Leeds, LS2 9JT, United Kingdom; 6 Children’s Acute Transport Service, Great Ormond Street Hospital, London, WC1N 3JH, United Kingdom; University Medical Center Rotterdam, NETHERLANDS

## Abstract

**Background:**

We determined the generalisability and cost-impact of adopting antibiotic-impregnated CVCs in all paediatric intensive care units (PICUs) in England, based on results from a large randomised controlled trial (the CATCH trial; ISRCTN34884569).

**Methods:**

BSI rates using standard CVCs were estimated through linkage of national PICU audit data (PICANet) with laboratory surveillance data. We estimated the number of BSI averted if PICUs switched from standard to antibiotic-impregnated CVCs by applying the CATCH trial rate-ratio (0.40; 95% CI 0.17,0.97) to the BSI rate using standard CVCs. The value of healthcare resources made available by averting one BSI as estimated from the trial economic analysis was £10,975; 95% CI -£2,801,£24,751.

**Results:**

The BSI rate using standard CVCs was 4.58 (95% CI 4.42,4.74) per 1000 CVC-days in 2012. Applying the rate-ratio gave 232 BSI averted using antibiotic CVCs. The additional cost of purchasing antibiotic-impregnated compared with standard CVCs was £36 for each child, corresponding to additional costs of £317,916 for an estimated 8831 CVCs required in PICUs in 2012. Based on 2012 BSI rates, management of BSI in PICUs cost £2.5 million annually (95% uncertainty interval: -£160,986, £5,603,005). The additional cost of antibiotic CVCs would be less than the value of resources associated with managing BSI in PICUs with standard BSI rates >1.2 per 1000 CVC-days.

**Conclusions:**

The cost of introducing antibiotic-impregnated CVCs is less than the cost associated with managing BSIs occurring with standard CVCs. The long-term benefits of preventing BSI could mean that antibiotic CVCs are cost-effective even in PICUs with extremely low BSI rates.

## Introduction

Bloodstream infection (BSI) is associated with serious adverse clinical outcome and increased costs in paediatric intensive care units (PICUs).[1–3] Central venous catheters (CVCs) are an important cause of BSI in this population.[4, 5] Results from a large, pragmatic randomised controlled trial (RCT) in PICU (the CATheter infections in CHildren, CATCH trial; ISRCTN34884569; http://www.nets.nihr.ac.uk/projects/hta/081347) showed that CVCs impregnated with antibiotics (minocycline and rifampicin) reduced BSI rates compared with standard CVCs.[6] However, guidelines for adults recommend using antibiotic-impregnated CVCs only for high-risk patients and there are no child-specific guidelines due to lack of RCTs in children until now.[7, 8] Prior to the CATCH trial, standard CVCs were used for the majority of children in UK PICUs.[7]

CATCH was the largest trial in PICU to date, recruiting 1485 children within 14 PICUs in 12 NHS Trusts in England, corresponding to 5% of all children admitted to PICUs in England and Wales during the trial period (2010–2012). If antibiotic-impregnated CVCs were adopted for children, it is likely that they would be bulk-purchased and used for all children requiring CVCs in PICU. Decisions on whether to purchase antibiotic-impregnated CVCs therefore need to take into account 1) the generalisability of trial results to all children who need a CVC, and 2) the overall budget-impact of purchasing the more expensive impregnated CVCs.

Firstly, in terms of generalisability, children recruited in CATCH might have different risks of BSI than children receiving impregnated CVCs outside the trial setting: Children in the trial were expected to require a CVC for three or more days, and would therefore have a higher risk of BSI than those staying for short periods of time; background BSI rates may now be lower than in the trial, as rates have been steadily decreasing over the past decade following the introduction of CVC care bundles and on-going improvements in infection control.[9, 10] Secondly, in terms of budget-impact, impregnated CVCs are approximately twice as expensive as standard CVCs, but these additional costs could be outweighed by the reduction in healthcare resources due to fewer BSIs with the antibiotic-impregnated CVCs.

We determined the generalisability of the CATCH trial by deriving BSI rates for all children expected to require CVCs, based on a linkage study using data from a number of PICUs across the NHS.[11] We determined the budget- and cost-impacts of adopting antibiotic-impregnated CVCs for all children expected to require a CVC in PICU by synthesising the following evidence:

1. the risk of BSI using standard CVCs (data linkage study)
2. the number of BSI potentially averted by using antibiotic-impregnated CVCs (CATCH trial results);
3. the additional costs of purchasing impregnated CVCs for all children (PICU survey data);
4. the value of the healthcare resources associated with each BSI (trial economic analysis).

## Methods

### Ethics, consent and permissions

The CATCH trial is registered on the ISRCTN registry (reference 34884569) and clinicaltrials.gov (NCT01029717). For PICANet, collection of personally identifiable data and specific permission for the data linkage study was approved by the Patient Information Advisory Group and the National Information Governance Board (now the NHS Health Research Authority Confidentiality Advisory Group) http://www.hra.nhs.uk/documents/2015/05/piag-register-8.xls. Ethics approval was granted by the Trent Medical Research Ethics Committee, ref. 05/MRE04/17 +5.

### Rate of BSI using standard CVCs

#### Data sources

Full details of the CATCH trial methodology are reported elsewhere.[6] The trial results gave a rate-ratio of 0.40 (95% CI 0.17, 0.97) comparing any BSI based on blood cultures taken between 48 hours after randomisation and 48 hours after CVC removal, for antibiotic versus standard CVCs.

The risk of BSI using standard CVCs in all children in PICU was derived by linking clinical records from the national PICANet database with national laboratory surveillance data coordinated by Public Health England.[12, 13] Details of the data linkage study have been published previously.[11] The resulting linked dataset captures approximately 71% of all children aged <16 years, admitted to 20 of the 25 PICUs in England and Wales between March 2003 and December 2012 and is broadly representative of the whole PICU population.[14]

For the present study, we restricted the linked dataset to children expected to require a standard CVC in PICUs in England. Types of CVCs used for emergency and elective admissions at each PICU were captured in responses to a PICU practice survey sent to a designated consultant at each PICU in 2009.[7] Where no response was obtained or the PICU was not included in the survey, we assumed that standard CVCs were used.

CVC use is not routinely captured for all admissions in PICANet, so we estimated the probability of CVC use for all admissions based on a subset of individual-level audit data from two hospitals, where CVC use was recorded. The subset of children most likely to have required a CVC was identified using multivariable logistic regression based on predictive variables in PICANet (use of vasoactive agents, length of stay and other clinical factors). BSI rates were then based on this subset of admissions. Full details of the predictive model are provided in S1 Appendix.

#### Case definition

We defined an episode of BSI as any positive blood culture isolated from a blood sample taken from two days after admission to two days after discharge from PICU. Repeated samples with positive cultures of the same organism within 14 days were treated as the same episode. We derived CVC days at risk by assuming that for CVCs were inserted at admission and removed at discharge from PICU.

#### Statistical analysis

Rates of BSI per 1000 CVC-days in CATCH and non-CATCH PICUs were modelled using multi-level Poisson regression. We accounted for clustering of admissions within PICUs by including a random effect for PICU. Appropriateness of the Poisson model was verified using a goodness-of-fit test based on the deviance statistic. For comparisons between units and over time, rates were adjusted for risk-factors identified as being significant (p<0.05). Likelihood-ratio tests were used to identify significant interactions between risk-factors.

### Number of BSI averted using antibiotic CVCs

The number of admissions requiring CVCs in all 23 PICUs in England was derived by combining the admission data in PICANet with PICU survey responses on the percentage of emergency and elective admissions requiring CVCs in 2012.[7] The BSI rate that would have occurred with antibiotic CVCs in place of standard CVCs was derived by applying the relative treatment effect (rate-ratio) for all BSI from the trial to the BSI rate using standard CVCs. This provided the excess number of BSI occurring with standard versus antibiotic CVCs.

### Budget-impact: additional costs of antibiotic CVCs

Antibiotic CVCs are more expensive than standard CVCs: £73 versus £42 for double lumen CVCs; £79 versus £43 for triple lumen CVCs. The total budget-impact of a policy to switch to antibiotic CVCs was calculated by multiplying the number of CVCs required by the maximum additional cost per CVC, i.e. £36. We assumed, conservatively, that any change in PICU length of stay, nursing or other resources would not impact on hospital budgets.

### Cost-impact: value of resources associated with managing BSI

Full details of the trial economics analysis are reported elsewhere. The difference in the 6-month risk-adjusted costs between patients who had a BSI versus those who did not was estimated as £10,975 per BSI (95% CI -£-2801 to £24,751). The total value of resources associated with managing BSI with standard CVCs was calculated by multiplying this value by the excess number of BSIs with standard versus antibiotic CVCs.

### Sensitivity analysis

We estimated the budget- and cost-impacts based on best and worst case scenarios for the total number of CVCs required and the excess number of BSIs with standard versus antibiotic CVCs. To account for uncertainty in estimates, we also performed probabilistic sensitivity analysis using Monte Carlo simulation. Values for each parameter were sampled from probability distributions based on observed data and 5000 iterations were performed to provide a 95% uncertainty interval for the cost-impact.[15]

## Results

### Rate of BSI using standard CVCs

Survey responses for the type of CVCs used prior to CATCH were obtained for 18 of the 23 PICUs in England (S1 Table). Only two PICUs reported not using standard CVCs for any admissions (both used heparin-bonded CVCs). The study sample contained admissions from the remaining 16 PICUs across England.

Of the 2488 admissions in the subset of CVC audit data, 1431 (58%) required a CVC. Applying the predictive model (S2 Table) to the linked dataset identified a subset of 21,381 admissions most likely to have received standard CVCs within the 16 PICUs between 2003–2012. Characteristics of these admissions (based on the full set of PICANet data) are provided in S3 Table.

**Fig 1:**
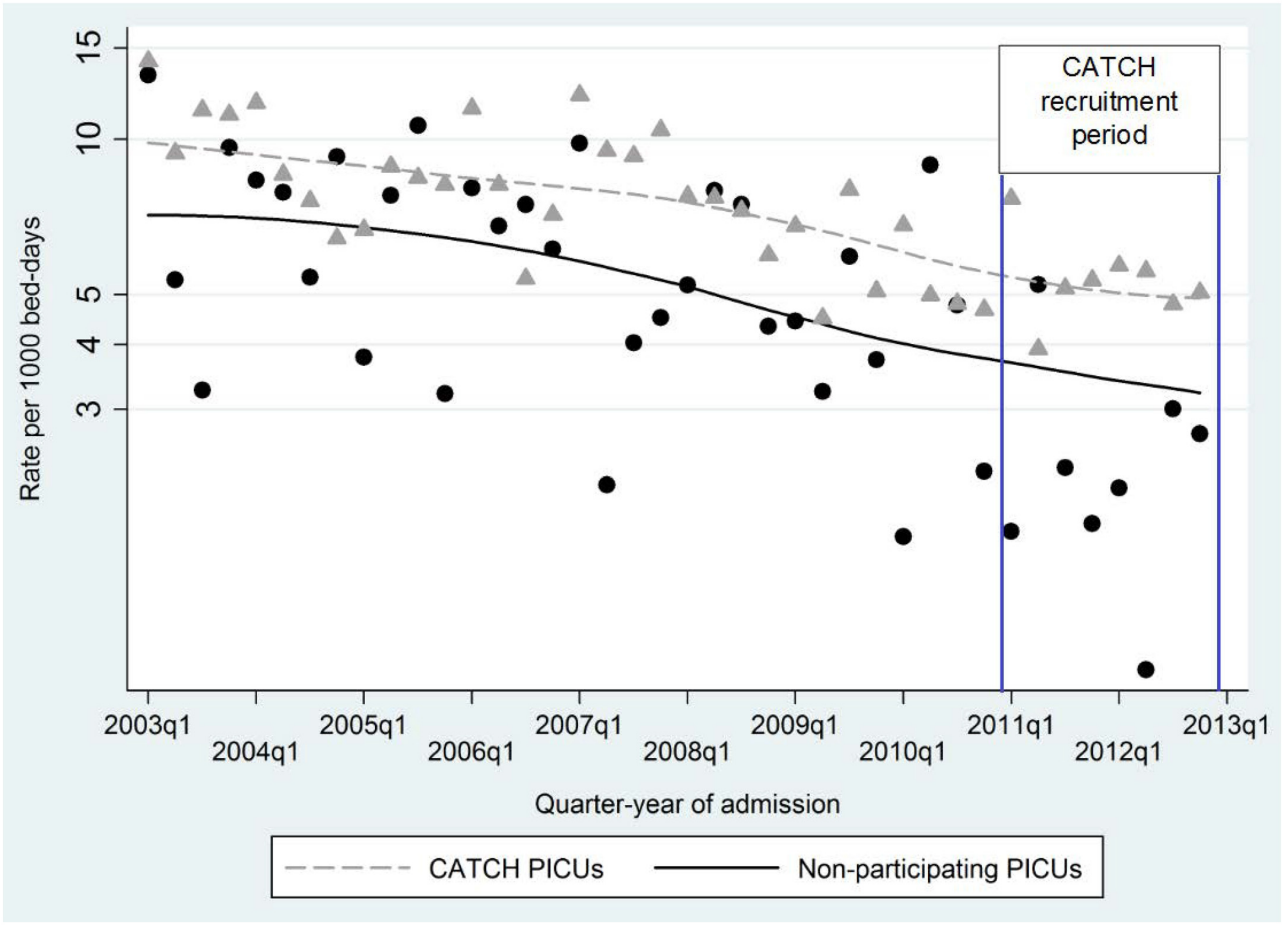
Risk-adjusted rates in bloodstream infection for children expected to have used standard central venous catheters in 16 PICUs in England; symbols = observed rates; lines = smoothed adjusted rates (log scale).

Risk-adjusted rates of BSI using standard CVCs decreased steadily between 2003 and 2012. Rates were greater for CATCH PICUs compared with non-participating PICUs (Fig 1). During 2012, 103/3021 (3.4%) of admissions experienced BSI, corresponding to a BSI rate using standard CVCs of 4.58 (95% CI 4.42, 4.74) per 1000 CVC-days (Table 1).

**Table 1:** Parameter estimates using in cost-impact analyses and sensitivity analysis.

Variable	Base case	Source	Sensitivity analysis
*BSI rate using standard CVCs*	4.58 (95% CI 4.42–4.74)	3021 admissions in 15 PICUs* 2012: Subset of admissions likely to have received standard CVCs identified by applying predictive model to linked dataset. Admissions identified by survey responses as receiving non-standard (heparin or antibiotic) CVCs were excluded.	Random sample taken with replacement from linked dataset, for the number of admissions expected to require CVCs.
*Rate ratio for antibiotic versus standard CVCs*	0.40 (95% CI 0.17–0.97)	Trial clinical effectiveness analyses	*Ln* N(-0.913, 0.415)
*BSI rate using antibiotic CVCs*	1.83; best case = 4.29; worst case = 0.81	Rate-ratio from the CATCH trial applied to BSI rate using standard CVCs in 2012	Derived from i) BSI rate using standard CVCs and ii) rate ratio
*Number of admissions requiring CVCs*	8831	Survey responses for the percentage of emergency (60%) and elective (50%) admissions requiring CVCs, applied to all admissions in PICANet in 2012 (15,739 admissions in 23 PICUs).	Emergency: Beta(60,40); Elective: Beta(50,50)
*Number of excess BSI with standard versus antibiotic CVCs*	232	BSI rates applied to CVC-days for admissions requiring CVCs in 2012	Derived from i) number of admissions requiring CVCs in 2012 and ii) estimated BSI rate using antibiotic CVCs
*Additional cost of antibiotic CVCs*	£36	Difference in costs between standard (£43) and antibiotic (£79) CVCs (conservative case assuming triple lumen CVCs used for all children)	Fixed at £36
*Costs associated with managing each BSI*	£10,975; (95% CI -£2,801, £24,751).	CATCH trial cost-effectiveness analysis	N(£10,975, £7,023)

* Data not available 2012 in 1 PICU

### Number of BSI averted using antibiotic CVCs

Survey responses indicated that on average, 60% of emergency admissions and 50% of elective admissions required CVCs (S1 Table). The number of children using CVCs in 2012 was estimated as 8831, corresponding to a total of 85,971 CVC-days. Applying the trial rate-ratio of 0.40 (95% CI 0.17, 0.97) for antibiotic versus standard CVCs gave an excess of 232 BSI with standard versus antibiotic CVCs in 2012, with best and worst case scenarios of 338 and 11 (Table 2).

**Table 2:** Cost-impact (value of resources made available) for a range of BSI rates and best and worst case scenarios for the effectiveness of antibiotic-impregnated CVCs. Bold indicates cost used in base case analysis.

					Cost-impact**	
	Rate ratio* for antibiotic versus standard CVCs	BSI per 1000 CVC-days using standard CVCs	Excess BSI with standard versus antibiotic CVCs	Lower limit: Cost per BSI: -£2801	Base case: Cost per BSI: £10,975	Upper limit: Cost per BSI: £24,751
***Base case***	0.40	4.58	232	-£648,606	**£2,541,397**	£5,731,401
***Worst case (upper CI)***	0.97	4.42	11	-£31,297	£122,631	£276,559
***Best case (lower CI)***	0.17	4.74	332	-£928,583	£3,638,415	£8,205,414
Hypothetical scenarios***						
	0.40	1.00	4	-£5,645	£22,119	£49,884
	0.40	2.00	101	-£11,290	£44,238	£99,767
	0.40	3.00	152	-£16,936	£66,358	£149,651
	0.40	4.00	202	-£22,581	£88,477	£199,534
	0.40	5.00	253	-£28,226	£110,596	£249,418
	0.40	6.00	303	-£33,871	£132,715	£299,301
	0.40	7.00	354	-£39,516	£154,834	£349,185
	0.40	8.00	405	-£45,161	£176,954	£399,069

* Estimated from the CATCH trial and trial economics analyses

** Positive values indicate the value of resources made available through averting BSI

*** Based on a typical PICU with 350 admissions per year

### Budget-impact: additional costs of antibiotic CVCs

Based on a CVC cost difference of £36, the cost to the NHS of purchasing antibiotic instead of standard CVCs in 2012 was 8831 x £36 = £317,916.

### Cost-impact: value of resources associated with managing BSI

**Fig 2:**
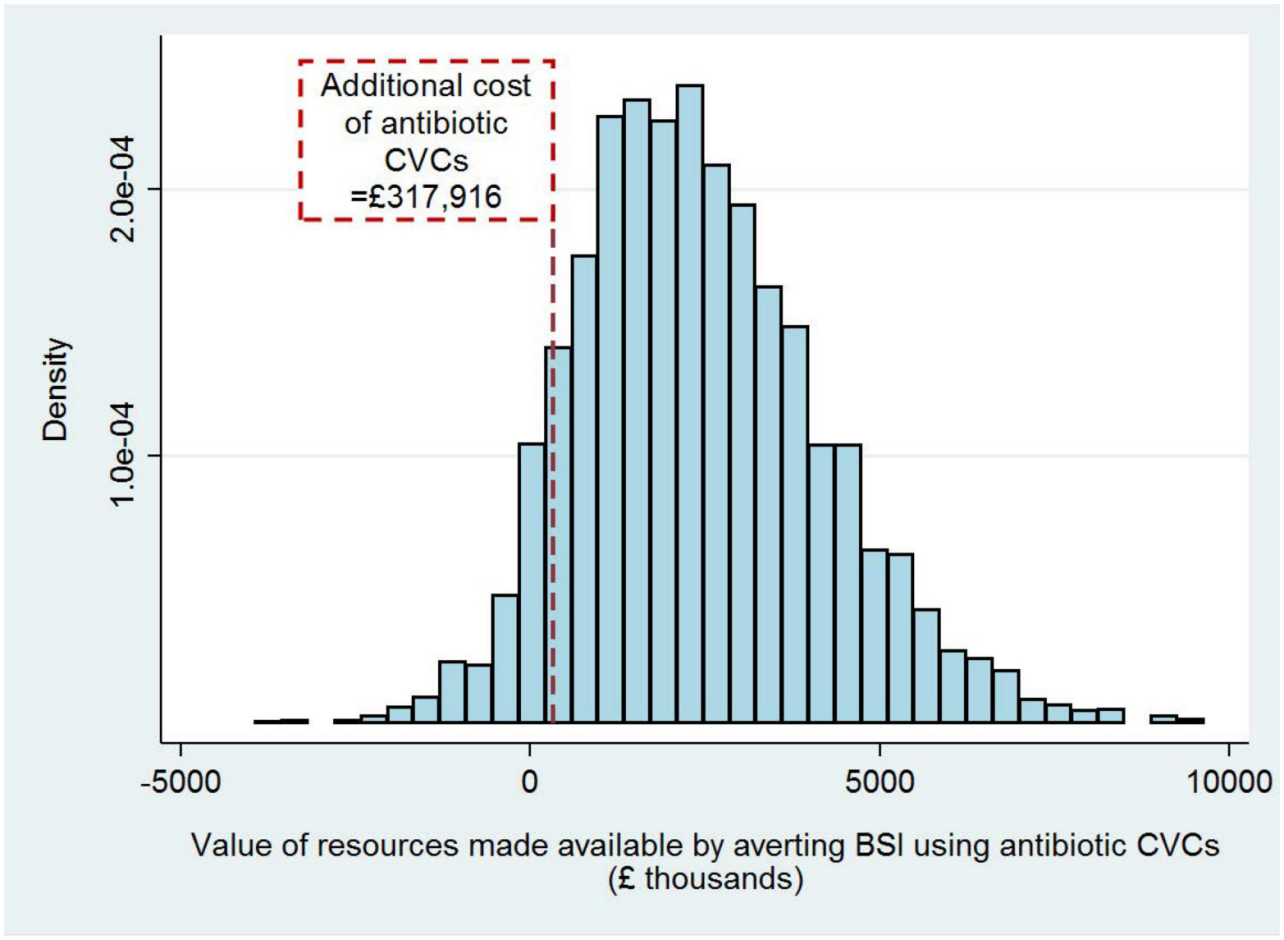
Probability distribution for the value of resources made available by averting BSI using antibiotic CVCs in all PICUs in England during 2012; 90% of the distribution represented costs greater than the additional cost of purchasing antibiotic CVCs.

Based on each BSI being associated with a mean cost of £10,975 (95% CI -£2,801, £24,751) over 6-months, the value of resources made available in 2012 through averting BSI with standard CVCs (i.e. the total costs of managing these BSIs) would have been 232 x £10,975 = £2,541,397. Best and worst case scenarios were -£925,583 and £8,205,414. The probabilistic sensitivity analysis provided a 95% uncertainty interval of -£66,544 to £5,557,451 for total resources made available through using antibiotic CVCs in 2012. There was a probability of 0.90 that the values of resources made available would be more than the additional costs of purchasing antibiotic CVCs (Fig 2).

**Fig 3:**
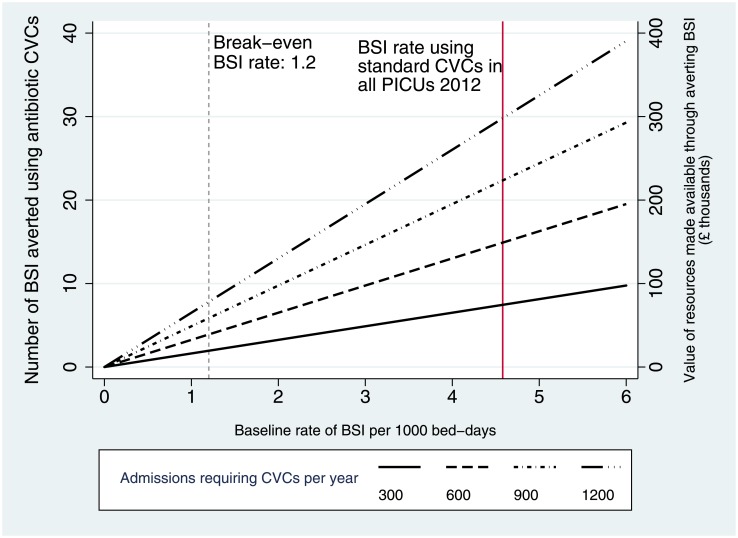
Cost-impact: Number of BSI averted and value of resources made available using antibiotic in place of standard CVCs for a range of baseline rates, assuming each BSI is associated with a mean cost of £10,975.

The estimated cost-impact for a typical PICU with 350 admissions per year is shown for a range of BSI rates in Table 2. Fig 3 shows that costs of purchasing antibiotic CVCs for all children who require them will be less than costs of managing BSI with standard CVCs for PICUs with BSI rates above 1.2 per 1000 bed-days.

## Discussion

Our study determined the generalisability of CATCH trial results and the cost-impact of changing practice in PICUs across England based on the trial results.[6] In terms of generalisability, observed rates of BSI using standard CVCs declined steadily over the past decade, including the period within which children were enrolled into the CATCH trial.[9, 16] Children receiving antibiotic CVCs now are therefore likely to have a lower risk of BSI than those participating in the CATCH trial.

In terms of budget-impact, antibiotic CVCs are more expensive than standard CVCs. However, antibiotic CVCs would likely be bulk-purchased for all children (including those with a lower risk of BSI than those participating in the trial). By estimating the number of BSI potentially averted using antibiotic CVCs for all children, we showed that the additional cost of purchasing antibiotic CVCs is lower than the value of resources associated with excess BSIs using standard CVCs.

We assumed that the relative treatment effect found in CATCH would be the same regardless of the baseline rate of BSI, i.e. that the effect would be the same for children who would have been ineligible for the trial because they were expected to stay <3 days in PICU. We reasoned that the biological mechanism through which antibiotic CVCs work is the same for low and high-risk patients (antibiotic-impregnated CVCs reduce the chance that bacteria track internally or externally along the CVC from the insertion site) and there was no a priori reason for an interaction. Randomised controlled trials of impregnated CVCs show similar results for long- and short-term CVCs, suggesting that effect is not modified in groups with different baseline risk or length of stay.[17] In CATCH, the event rate was low and there was limited power to assess variation in the treatment effect according to the duration of CVC. In reality, 72% of children recruited in CATCH required a CVC for 3 or more days.

There are a limitations to this study. Firstly, although Public Health England request that only clinically significant isolates are reported to the national surveillance system, the data used in the linkage study could have included BSI that should have been considered as contaminants. Secondly, BSI rates using standard CVCs were estimated using a predictive model as it was not possible to identify which children in PICANet had a CVC. We also relied on the assumption that for children likely to have required CVCs, CVCs would remain in place for the entire PICU stay. There is no clear direction of bias as we may have over- or under-estimated CVC-days, but our assumptions are reasonable based on the subset of CATCH participants. Finally, we relied on survey responses to derive the number of CVCs required in PICU, but we addressed this and uncertainty in other parameter estimates by performing sensitivity analyses.[18, 19]

## Conclusion

Our results suggest that the benefits of using antibiotic-impregnated CVCs apply even for PICUs with low rates of BSI. These finding are consistent with systematic review evidence on the cost-effectiveness of impregnated CVCs in adults, which indicates that implementation of impregnated CVCs would be cost-effective for a range of relative risks and for baseline incidence of BSI as low as 0.2%.[20] CATCH is the first trial to assess the effectiveness of antibiotic-impregnated versus standard CVCs in children, and the results of this generalisability study add to strong evidence in adults. Furthermore, as our cost estimates only consider use of hospital resources, the true cost of BSI and the benefits of antibiotic CVCs may be even greater when longer term outcomes of BSI are taken into account.

## Supporting Information

S1 Appendix Predictive model identifying children most likely to require CVCs in PICU. (DOC)

S1 Table Survey results on type of CVC used prior to CATCH and percentage of admissions requiring CVCs. Shaded boxes correspond to the 12 NHS Trusts participating in CATCH (14 PICUs). *no data in linked dataset.
(DOCX)

S2 Table Independent predictors of central venous catheter use in CVC audit data (basis for the predictive model). (DOCX)

S3 Table Characteristics of admissions during the 23-month trial period (December 2010 to November 2012) in all PICUs in England. (DOCX)

## References

[1] Abou Elella R, Najm H, Balkhy H, Bullard L, Kabbani M. Impact of bloodstream infection on the outcome of children undergoing cardiac surgery. Pediatr Cardiol. 2010;31(4):483–9. 10.1007/s00246-009-9624-x 20063161

[2] Elward AM, Hollenbeak CS, Warren DK, Fraser VJ. Attributable cost of nosocomial primary bloodstream infection in pediatric intensive care unit patients. Pediatrics. 2005;115(4):868–72. 15805357 10.1542/peds.2004-0256

[3] Nowak JE, Brilli RJ, Lake MR, Sparling KW, Butcher J, Schulte M, et al Reducing catheter-associated bloodstream infections in the pediatric intensive care unit: Business case for quality improvement. Pediatr Crit Care Med. 2010;11(5):579–87. 10.1097/PCC.0b013e3181d90569 20308931

[4] Lakshmi KS, Jayashree M, Singhi S, Ray P. Study of nosocomial primary bloodstream infections in a Pediatric Intensive Care Unit. J Trop Pediatr. 2007;53(2):87–92. 10.1093/tropej/fml073 17151083

[5] Yogaraj JS, Elward AM, Fraser VJ. Rate, risk factors, and outcomes of nosocomial primary bloodstream infection in pediatric intensive care unit patients. Pediatrics. 2002;110(3):481–5. 12205248 10.1542/peds.110.3.481

[6] Gilbert R, Mok Q, Dwan K, Harron K, Moitt T, Millar M, et al Pragmatic randomised, controlled trial of impregnated central venous catheters for preventing bloodstream infection in children. Lancet. 2016;in press. 10.1016/S0140-6736(16)00340-8 26946925

[7] Harron K, Ramachandra G, Mok Q, Gilbert R. Consistency between guidelines and reported practice for reducing the risk of catheter-related infection in British paediatric intensive care units. Intens Care Med. 2011;37(10):1641–7. 10.1007/s00134-011-2343-9 21877212

[8] Department of Health. Saving Lives: reducing infection, delivering clean and safe care. Department of Health, London 2007.

[9] Harron K, Wade A, Muller-Pebody B, Goldstein H, Parslow R, Gray J, et al Risk-adjusted monitoring of blood-stream infection in paediatric intensive care: a data linkage study. Intens Care Med. 2013;39(6):1080–7. 10.1007/s00134-013-2841-z 23404472

[10] Harron K, Parslow R, Mok Q, Tibby S, Wade A, Muller-Pebody B, et al Monitoring quality of care through linkage of administrative data: national trends in bloodstream infection in UK paediatric intensive care units 2003–2012. Crit Care Med. 2015;in press. 10.1097/CCM.0000000000000941 25746506

[11] Harron K, Goldstein H, Wade A, Muller-Pebody B, Parslow R, Gilbert R. Linkage, evaluation and analysis of national electronic healthcare data: application to providing enhanced blood-stream infection surveillance in paediatric intensive care. PLoS One. 2013;8(12):e85278 10.1371/journal.pone.0085278 24376874 PMC3869925

[12] Universities of Leeds and Leicester. Paediatric Intensive Care Audit Network National Report 2011–2013. 2013.

[13] Wilson J, Elgohari S, Livermore DM, Cookson B, Johnson A, Lamagni T, et al Trends among pathogens reported as causing bacteraemia in England, 2004–2008. Clin Microbiol Infect. 2011;17(3):451–8. 10.1111/j.1469-0691.2010.03262.x 20491834

[14] Harron K. Evaluating data linkage techniques for the analysis of bloodstream infection in paediatric intensive care: University College London; 2014.

[15] O’Hagan A, McCabe C, Akehurst R, Brennan A, Briggs A, Claxton K, et al Incorporation of uncertainty in health economic modelling studies. Pharmacoeconomics. 2005;23(6):529–36. 10.2165/00019053-200523060-00001 15960550

[16] Harron K, Mok Q, Parslow R, Muller-Pebody B, Gilbert R, Ramnarayan P. Risk of bloodstream infection in children admitted to paediatric intensive care units in England and Wales following emergency inter-hospital transfer. Intens Care Med. 2014;40(12):1916–23. 10.1007/s00134-014-3516-0 PMC4239794 25331585

[17] Gilbert R, Harden M. Effectiveness of impregnated central venous catheters for catheter related blood stream infection: a systematic review. Curr Opin Infect Dis. 2008;21(3):235–45. 10.1097/QCO.0b013e3282ffd6e0 18448967

[18] Briggs A. Probabilistic analysis of cost-effectiveness models: statistical representation of parameter uncertainty. Value Health. 2005;8(1):1–2. 10.1111/j.1524-4733.2005.08101.x 15841888

[19] Claxton K, Sculpher M, McCabe C, Briggs A, Akehurst R, Buxton M, et al Probabilistic sensitivity analysis for NICE technology assessment: not an optional extra. Health Econ. 2005;14(4):339–47. 10.1002/hec.985 15736142

[20] Hockenhull J, Dwan K, Boland A, Smith G, Bagust A, Dündar Y, et al The clinical effectiveness and cost-effectiveness of central venous catheters treated with anti-infective agents in preventing bloodstream infections: a systematic review and economic evaluation. Health Technol Asses. 2008;12(12):1–154. 10.3310/hta12120 18405471

