# Supplementary material for: Generalisability and Cost-Impact of Antibiotic-Impregnated Central Venous Catheters for Reducing Risk of Bloodstream Infection in Paediatric Intensive Care Units in England
Source: PLoS One. 2016 Mar 21;11(3):e0151348. doi: 10.1371/journal.pone.0151348 (PMC4801221; doi:10.1371/journal.pone.0151348)
Supplement: S1 Appendix — (DOC) [file pone.0151348.s001.doc]

# S1 Appendix: Predictive model identifying children most likely to require CVCs in PICU

PICANet does not record insertion or removal of central venous catheters (CVCs). However, through the use of CVC audit data from two paediatric intensive care units (PICUs), it was possible to create a predictive model to identify admissions most likely to have required a CVC in PICANet.

### **Methods**

CVC audit data

CVC audit data were obtained from four PICUs. Data from PICUs 1 and 2 comprised individual-level information, and data from PICUs 3 and 4 comprised aggregate data. At PICU 1, the insertion and duration of CVCs were recorded for six months between July and December 2009. At PICU 2, the number of CVCs present for each patient was recorded on a daily basis between December 2005 and March 2012.

At PICU 3, the total number of patients admitted and the number of patients with one or more CVCs was recorded by month between January 2011 and February 2012. At PICU 4, the total number of patients admitted to PICU, the number of patients with one or more CVCs and the total number of CVCs in place were recorded each day between December 2009 and June 2012.

A predictive model for CVC use

CVC use was identified within PICANet using PICANet ID and hospital number from the audit data. Multivariable logistic regression was then used to model the probability of CVC use dependent on a set of predictors:


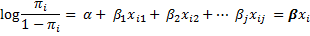


where
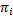
 is the probability of CVC use for patient *i*, *α* is the constant term and
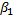
…
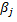
are the set of predictors. To identify the best-fitting set of predictors, all possible regression models were tested, ranging from the model including only the intercept to the model including all possible predictor variables. Models were compared using the Bayesian Information Criterion (BIC).

Evaluating the performance of the predictive model

To quantify the performance of a predictive model, two measures are typically used:

- Discrimination (the ability of predicted probabilities to correctly classify children by CVC use)
- Calibration (agreement between observed CVC use and predicted probability of CVC use)

To measure the discrimination of the predictive model, the c-index (equivalent to the area under the receiver operator characteristic (ROC) curve) was calculated. The c-index corresponds to the chance that the predicted probability of CVC use in someone who did require a CVC is greater than the predicted probability of CVC use in someone who did not require a CVC. The greater the c-index, the more discriminative the model.

To measure the calibration of the predictive model, observed CVC use and predicted probabilities were compared using the calibration slope (or linear predictor), as described in Steyerberg et al. The calibration slope is the regression coefficient β in the logistic regression of observed CVC use (binary variable) with predicted CVC use (probability) as the only predictor. Predicted CVC use is calculated as the linear combination of regression coefficients as estimated from the predictive model. In a perfectly calibrated model, the regression coefficient β in the following model would be equal to 1:
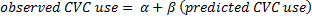


By definition, when the model is developed and tested in the same sample, the calibration slope will be equal to 1. However when predictive models are tested in new data, the calibration slope is often <1, since most models provide predictions that are too extreme. The closer the β coefficient to 1, the better the calibration of the model.

Internal validation

When predictive models are derived and tested within the same sample of data, measures of predictive ability (e.g. calibration/discrimination) are likely to be over-optimistic. This is due to model over-fitting, where the ‘apparent’ performance in the model derivation dataset is likely to be better than the performance in a new set of data. Bootstrapping is an approach which addresses this problem and makes use of all available data, producing more stable results. The method involves repeatedly sampling from the original data, a process which simulates sampling from the underlying population from which the original data were drawn. An estimate of the ‘optimism’ in the predictive ability of the model is made by comparing model performance in bootstrap samples with ‘apparent’ performance in the derivation sample.

Bootstrapping was used to estimate the optimism in the predictive ability of the model as measured in the derivation sample of data (i.e. the ‘apparent’ performance in the CVC audit data). The optimism reflects the difference between model performance in the derivation dataset, and the performance of the model in a separate but similar dataset drawn from the same underlying population. Optimism was estimated as the difference between the apparent performance of a model derived in a bootstrap sample and ‘test’ performance when the same model was applied to the derivation sample (CVC audit data).

Finally, apparent model performance (as measured in the derivation sample) was adjusted for optimism/over-fitting by subtracting the estimate of optimism from the measure of predictive ability (calibration slope or c-index). The resulting measure of performance is said to be ‘internally validated’.

Choosing a probability cut-off

A probability cut-off is required to classify children as either requiring a CVC or not. Higher probability cut-offs result in greater specificity; lower probability cut-offs results in greater sensitivity. A visualisation of the trade-off between sensitivity and specificity was provided by the receiver operator characteristic (ROC) curve. Two main criteria are used for finding the optimal cut-off based on maximising the area under the ROC curve and probability cut-offs according to both these criteria were calculated:

1. The minimum distance criteria assumes that the optimal cut-off minimises the distance between the point (0,1) and the ROC curve, i.e. the minimal value of:


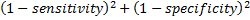


1. The Youden index criteria assumes that the optimal cut-off maximises the vertical distance between the ROC curve and the line of equality where sensitivity=1-specificity, i.e. the maximum value of:


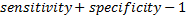


External validation

Aggregate CVC audit data from PICUs 3 and 4 were not used for development of the predictive model (individual-level data were not available) but could provide estimates of the average proportion of children requiring a CVC in PICU. To externally validate the predictive model, the actual number of admissions and bed-days with CVCs in the audit data from PICUs 3 and 4 were compared to the results of the predictive model (with shrinkage factor applied).

### **Results**

A predictive model for CVC use

The best fitting model included length of stay, vasoactive agent, admission from ward, renal support and invasive ventilation. No significant first-order interactions were found. The predicted probability of CVC use for each admission
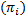
 was derived from the logistic model:


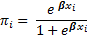


where
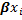
 was the linear predictor of the BIC model.

Evaluating the performance of the predictive model

Discrimination

The c-index of the predictive model in the original sample was estimated as 0.778. The average c-index in 100 bootstrap samples was 0.778 and on average, the c-index as measured in the derivation sample was 0.004 higher than when measured in the test sample. Subtracting this estimate of optimism from the apparent performance in the derivation sample produced an internally validated c-index of 0.778-0.004=0.774. This indicated that the model performed reasonably well at classifying children as requiring a CVC.

Calibration

By definition, the calibration slope (β coefficient) for the regression of observed CVC use and predicted CVC use in the original sample was 1, as the model was developed and tested in the same sample:


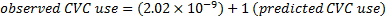


The average calibration slope in 100 bootstrap samples was 0.967 and on the average, the calibration slope in the derivation dataset was 0.033 higher than when measured in the test dataset. Subtracting this estimate of optimism from the apparent performance in the derivation sample produced an internally validated calibration slope of 1-0.033=0.967. This indicated close agreement between observed CVC use and CVC use as predicted in the model.

The coefficients in the original model were multiplied by the shrinkage factor of 0.967 to provide a final model, adjusted for over-fitting.

Choosing a probability cut-off

The Youden index indicated that the optimal probability cut-off was 0.57. With this cut-off, the sensitivity of the predictive model for capturing admissions requiring a CVC was 61%; specificity was 82%; positive predictive value was 82% and negative predictive value was 61%.

External validation

Compared with the aggregate CVC data, the model predicted that 54.6% and 63.5% of admissions in Newcastle PICU and Birmingham PICU required a CVC, compared with true values of 49.4% and 54.6% respectively. The predictive model identified 80% of the CATCH admissions as having a CVC.

**References**

1. Steyerberg EW, Harrell Jr FE, Borsboom GJJM, Eijkemans MJC, Vergouwe Y, Habbema JDF: **Internal validation of predictive models: efficiency of some procedures for logistic regression analysis**. *J Clin Epidemiol* 2001, **54**(8):774-781.

2. Harrell F, Lee KL, Mark DB: **Tutorial in biostatistics multivariable prognostic models: issues in developing models, evaluating assumptions and adequacy, and measuring and reducing errors**. *Stat Med* 1996, **15**:361-387.

3. Steyerberg EW, Eijkemans MJC, Harrell FE, Habbema JDF: **Prognostic modeling with logistic regression analysis: in search of a sensible strategy in small data sets**. *Med Decis Making* 2001, **21**(1):45-56.

4. Akobeng AK: **Understanding diagnostic tests 3: receiver operating characteristic curves**. *Acta Pædiatrica* 2007, **96**(5):644-647.

5. Fluss R, Faraggi D, Reiser B: **Estimation of the Youden Index and its associated cutoff point**. *Biom J* 2005, **47**(4):458-472.
