# Supplementary material for: Generalisability and Cost-Impact of Antibiotic-Impregnated Central Venous Catheters for Reducing Risk of Bloodstream Infection in Paediatric Intensive Care Units in England
Source: PLoS One. 2016 Mar 21;11(3):e0151348. doi: 10.1371/journal.pone.0151348 (PMC4801221; doi:10.1371/journal.pone.0151348)
Supplement: S1 Table — Shaded boxes correspond to the 12 NHS Trusts participating in CATCH (14 PICUs). *no data in linked dataset. (DOCX) [file pone.0151348.s002.docx]

**S1 Table: Survey results on type of CVC used prior to CATCH and percentage of admissions requiring CVCs. Shaded boxes correspond to the 12 NHS Trusts participating in CATCH (14 PICUs). *no data in linked dataset**

|  | **Type of CVC used prior to CATCH**  **2009 survey** | | | **Admissions requiring a CVC**  **2012 survey** | |
| --- | --- | --- | --- | --- | --- |
| **PICU Trust** | **Emergency admissions (%)** | **Elective admissions (%)** | **Emergency admissions (%)** | | **Elective admissions (%)** |
| **1** | *not surveyed* | *not surveyed* | 75 | | 25 |
| **2** | standard/heparin | standard/heparin | 57 | | 93 |
| **3** | *not surveyed* | *not surveyed* | <5 | | <5 |
| **4** | standard/heparin | standard | 80 | | 90 |
| **5** | standard | standard | 85 | | 50 |
| **6** | standard | standard | no response | | no response |
| **7** | standard/antibiotic | standard | 60-100 | | 60-90 |
| **8*** | standard | standard | 80 | | 80 |
| **9*** | heparin | heparin | 60 | | 30 |
| **10** | standard | standard | 30 | | 50 |
| **11** | standard | standard | 50 | | 50 |
| **12** | standard | standard | no response | | no response |
| **13** | standard | standard | no response | | no response |
| **14** | standard | standard | 75 | | no response |
| **15** | heparin | heparin | 87 | | 18 |
| **16** | standard | standard | 60 | | 20 |
| **17** | standard | standard | 70 | | 80 |
| **18** | standard | standard | no response | | no response |
| **19** | no response | no response | no response | | no response |
| **20** | standard | antibiotic | 50 | | 50 |
| **21** | heparin | standard/heparin | 50 | | 30 |
| **22*** | *not surveyed* | *not surveyed* | no response | | no response |
| **23*** | *not surveyed* | *not surveyed* | 60 | | 50 |
| **Average** |  |  | **60** | | **50** |
