# Supplementary material for: Generalisability and Cost-Impact of Antibiotic-Impregnated Central Venous Catheters for Reducing Risk of Bloodstream Infection in Paediatric Intensive Care Units in England
Source: PLoS One. 2016 Mar 21;11(3):e0151348. doi: 10.1371/journal.pone.0151348 (PMC4801221; doi:10.1371/journal.pone.0151348)
Supplement: S2 Table — (DOCX) [file pone.0151348.s003.docx]

**S2 Table: Independent predictors of central venous catheter use in CVC audit data (basis for the predictive model)**

|  | **Odds ratio**  **(95% confidence interval)** | **p-value** |
| --- | --- | --- |
| Length of stay (hours) | 1.003 (1.000,1.004) | <0.0001 |
| Vasoactive agent | 4.443 (3.600,5.513) | <0.0001 |
| Admission from ward | 1.428 (1.200,1.738) | <0.0001 |
| Renal support | 3.952 (2.000,7.822) | <0.0001 |
| No ventilation or non-invasive only | 1 |  |
| Invasive ventilation | 2.547 (1.900,3.350) | <0.0001 |
| Invasive and non-invasive ventilation | 2.278 (1.500,3.395) | <0.0001 |
