# Supplementary material for: Generalisability and Cost-Impact of Antibiotic-Impregnated Central Venous Catheters for Reducing Risk of Bloodstream Infection in Paediatric Intensive Care Units in England
Source: PLoS One. 2016 Mar 21;11(3):e0151348. doi: 10.1371/journal.pone.0151348 (PMC4801221; doi:10.1371/journal.pone.0151348)
Supplement: S3 Table — (DOCX) [file pone.0151348.s004.docx]

**S3 Table: Characteristics of admissions during the 23-month trial period (December 2010 to November 2012) in all PICUs in England**

|  |  | **CATCH participants* (n=1398)** | | **Admissions expected to require CVCs** (n=20,199)** | | **All admissions during trial period (n=53,897)** | |
| --- | --- | --- | --- | --- | --- | --- | --- |
|  |  | n | % | n | % | n | % |
| **Length of stay (hours)** | 1-<4 | 3 | 0.2 | 186 | 0.9 | 1,482 | 2.7 |
|  | 4-<12 | 18 | 1.3 | 438 | 2.2 | 3,764 | 7.0 |
|  | 12-<24 | 101 | 7.2 | 1,699 | 8.4 | 9,647 | 17.9 |
|  | 24-<48 | 175 | 12.5 | 2,959 | 14.6 | 10,919 | 20.3 |
|  | 48+ | 1,101 | 78.8 | 14,917 | 73.9 | 28,085 | 52.1 |
| **Age (years)** | <1 | 815 | 58.3 | 11,775 | 58.3 | 27,323 | 50.7 |
|  | 1-4 | 327 | 23.4 | 4,473 | 22.1 | 13,405 | 24.9 |
|  | 5-10 | 144 | 10.3 | 2,023 | 10.0 | 6,837 | 12.7 |
|  | 11-15 | 112 | 8.0 | 1,926 | 9.5 | 6,328 | 11.7 |
|  | Unknown |  | 0.0 | 2 | 0.0 | 4 | 0.0 |
| **Vasoactive agents** | | 1,054 | 75.4 | 17,081 | 84.6 | 18,792 | 34.9 |
| **Renal support** |  | 148 | 10.6 | 1,469 | 7.3 | 1684 | 3.1 |
| **Paediatric Index of Mortality** | <1% | 150 | 10.7 | 1,857 | 9.2 | 13,855 | 25.7 |
|  | 1-5% | 744 | 53.2 | 10,332 | 51.2 | 25,840 | 47.9 |
|  | 5-15% | 354 | 25.3 | 5,472 | 27.1 | 10,520 | 19.5 |
|  | 15-30% | 103 | 7.4 | 1,486 | 7.4 | 2290 | 4.2 |
|  | 30%+ | 47 | 3.4 | 1,052 | 5.2 | 1392 | 2.6 |
| **Ventilation status** | Neither | 33 | 2.4 | 442 | 2.2 | 12,652 | 23.5 |
|  | Non-invasive only | 10 | 0.7 | 159 | 0.8 | 2,620 | 4.9 |
|  | Invasive only | 1,017 | 72.7 | 16,170 | 80.1 | 32,882 | 61.0 |
|  | Both | 337 | 24.1 | 3,424 | 17.0 | 5,625 | 10.4 |
|  | Unknown | 1 | 0.1 | 4 | 0.0 | 118 | 0.2 |
| **Type of admission** | Planned | 572 | 40.9 | 9,015 | 44.6 | 21,844 | 40.5 |
|  | Unplanned | 826 | 59.1 | 11,180 | 55.3 | 31,992 | 59.4 |
|  | Unknown |  | 0.0 | 4 | 0.0 | 61 | 0.1 |
| **Source of admission** | Same hospital | 729 | 52.1 | 11,713 | 58.0 | 32,966 | 61.2 |
|  | Other hospital | 667 | 47.7 | 8,374 | 41.5 | 20,210 | 37.5 |
|  | Unknown | 2 | 0.1 | 112 | 0.6 | 721 | 1.3 |
| **Primary diagnosis at admission** | Cardio | 707 | 50.6 | 10,687 | 52.9 | 16,818 | 31.2 |
|  | Respiratory | 273 | 19.5 | 3,751 | 18.6 | 14,295 | 26.5 |
|  | Infection | 100 | 7.2 | 1,078 | 5.3 | 2,333 | 4.3 |
|  | Other | 318 | 22.7 | 4,683 | 23.2 | 20,451 | 37.9 |
| **Care area of admission** | A & E | 242 | 17.3 | 2,379 | 11.8 | 9,422 | 17.5 |
|  | HDU | 72 | 5.2 | 878 | 4.3 | 2,410 | 4.5 |
|  | ICU / PICU / NICU | 222 | 15.9 | 3,802 | 18.8 | 8,112 | 15.1 |
|  | Other intermediate care area | 8 | 0.6 | 466 | 2.3 | 1315 | 2.4 |
|  | Recovery only | 3 | 0.2 | 39 | 0.2 | 155 | 0.3 |
|  | Theatre and recovery | 565 | 40.4 | 8,422 | 41.7 | 20,566 | 38.2 |
|  | Unknown | 9 | 0.6 | 165 | 0.8 | 919 | 1.7 |
|  | Ward | 273 | 19.5 | 3,889 | 19.3 | 10,417 | 19.3 |
|  | X-ray / endoscopy / CT | 4 | 0.3 | 159 | 0.8 | 581 | 1.1 |
| **Retrieval** | Yes | 596 | 42.6 | 7,464 | 37.0 | 18,230 | 33.8 |
| **Retrieval team** | Non-specialist team | 19 | 1.4 | 721 | 3.6 | 2,031 | 3.8 |
|  | Other specialist team | 306 | 21.9 | 4,358 | 21.6 | 9,681 | 18.0 |
|  | Own team | 263 | 18.8 | 2,339 | 11.6 | 6,388 | 11.9 |
|  | Unknown | 8 | 0.6 | 46 | 0.2 | 130 | 0.2 |
| **Sex** | Male | 811 | 58.0 | 11,363 | 56.3 | 30,428 | 56.5 |
|  | Female | 587 | 42.0 | 8,830 | 43.7 | 23,449 | 43.5 |
|  | Unknown |  | 0.0 | 6 | 0.0 | 20 | 0.0 |
| **PICU type** | General | 59 | 4.2 | 2,831 | 14.0 | 15,828 | 29.4 |
|  | Mixed | 1,286 | 92.0 | 16,997 | 84.1 | 37,386 | 69.4 |
|  | Cardiac | 53 | 3.8 | 371 | 1.8 | 683 | 1.3 |
| **PICU size (admissions per year)** | <650 | 59 | 4.2 | 2,373 | 11.7 | 14,255 | 26.4 |
|  | 650-1000 | 620 | 44.3 | 3,227 | 16.0 | 8,731 | 16.2 |
|  | >1000 | 719 | 51.4 | 14,599 | 72.3 | 30,911 | 57.4 |

*consenting to linkage with PICANet

** based on predictive model
